# Ratio of triglyceride to high-density lipoprotein cholesterol and risk of major cardiovascular events in kidney transplant recipients

**DOI:** 10.1007/s10157-019-01776-9

**Published:** 2019-08-29

**Authors:** Ji Eun Kim, Mi-Yeon Yu, Yong Chul Kim, Sang-il Min, Jongwon Ha, Jung Pyo Lee, Dong Ki Kim, Kook-Hwan Oh, Kwon-Wook Joo, Curie Ahn, Yon Su Kim, Hajeong Lee

**Affiliations:** 1 Department of Internal Medicine, Seoul National University College of Medicine, 103 Daehakro, Jongno-gu, Seoul, 03080 South Korea; 2 Department of Internal Medicine, Hanyang University Guri Hospital, Guri, South Korea; 3 Department of Surgery, Seoul National University Transplantation Research Laboratory, Seoul National University Hospital, Seoul, South Korea; 4 Department of Internal Medicine, Seoul National University Boramae Medical Center, Seoul, Korea; 5 Kidney Research Institute, Seoul National University College of Medicine, Seoul, South Korea

**Keywords:** Cholesterol, Cardiovascular, TG/HDL-C, Statins, Kidney transplantation

## Abstract

**Background:**

Dyslipidemia is common in kidney transplant (KT) recipients. We analyzed the ratio of triglyceride to high-density lipoprotein cholesterol (TG/HDL-C) in KT recipients to identify risk factors for major cardiovascular events (MACE).

**Methods:**

We retrospectively included KT recipients with a lipid profile performed 1 year after transplantation. We classified patients according to the TG/HDL-C divided into quintiles. Subsequently, we analyzed the association between TG/HDL-C and MACE, defined as heart failure, coronary artery disease, and cerebrovascular disease confirmed by imaging studies.

**Results:**

A total of 1301 KT recipients were enrolled. The median follow-up duration was 7.4 years (interquartile range 4.4–11.1 years). During the follow-up period, 80 (6.2%) patients developed MACE, which included 38 of unstable anginas, 9 of MIs, 19 of heart failures, 18 of cerebral infarcts, and 4 of cerebral hemorrhages. The fourth and fifth quintiles of TG/HDL-C showed a significantly increased risk of MACE [fourth quintile: adjusted hazard ratio (aHR), 3.38; 95% confidence interval (CI) 1.44–7.95; *p* = 0.005, fifth quintile: aHR, 2.67; 95% CI 1.13–6.30; *p* = 0.02]) compared to the second quintile of TG/HDL-C. This association is particularly evident in subgroups of non-DM, HTN, no history of CVD, and statin users.

**Conclusions:**

Higher TG/HDL-C levels may be associated with MACE risk in KT recipients.

## Introduction

Patients with end-stage renal disease (ESRD) have up to a 30-fold increased cardiovascular (CV) risk compared with the general population [1, 2]. In kidney transplant (KT) recipients, cardiovascular disease (CVD) mortality rates are significantly lower than that in an age-stratified dialysis population, suggesting that renal transplantation reduces CV risk in patients with ESRD [3, 4]. However, the incidence of CVD in KT recipients remains 3–5 times higher than that of the general population [3, 5]. CVD accounts for 35–50% of all-cause mortality in KT recipients and is considered the predominant cause of death [4, 6].

One of the most important CVD risk factors is dyslipidemia [7, 8]. Dyslipidemia is estimated to be present in up to 80% of KT recipients [9], which is similar to the prevalence of dyslipidemia in patients with ESRD prior to transplantation [10]. Dyslipidemia in ESRD is known to be associated with inflammation, changes in lecithin–cholesterol acyltransferase enzyme activity, and decreased insulin sensitivity [11]. After transplantation and renal function recovery, lipid disturbances typically persist, but exhibit a different profile because of the varying effects of immunosuppressive drugs [12]. Among immunosuppressive agents, corticosteroids, cyclosporine, and mammalian targets of rapamycin inhibitors are associated with elevated lipid levels [13]. The dyslipidemia-related factors in ESRD are partially, but not completely, reversible, and the use of immunosuppressive agents, diet, obesity, and genetic predisposition are all thought to contribute to the prevalence of dyslipidemia in transplant patients [13, 14]. The role of dyslipidemia in elevating CV risk in KT recipients is not clearly defined [13]. However, some studies have suggested that the treatment of dyslipidemia could decrease CV risk in KT recipients [15, 16].

Dyslipidemia is defined as an overall change in the lipid profile rather than an elevation in only the total or low-density lipoprotein cholesterol (LDL-C) levels [17]. Triglycerides (TG), LDL-C, and high-density lipoprotein cholesterol (HDL-C) are closely related molecules in terms of their metabolism and effects on multiple organs [18]. Recent studies demonstrated that the ratios of lipid components, such as total cholesterol/HDL-C and TG/HDL-C, are better indicators of CV risk than their individual levels [19-22]. Among these, the ratio of TG to HDL-C (TG/HDL-C) has been shown to be a strong predictor of myocardial infarction (MI), LDL phenotype B, and atherogenic risk [22-24]. In this study, we investigated the association between TG/HDL-C and the development of major adverse cardiovascular events (MACE) after renal transplantation.

## Methods

### Study design and population

The study design was approved by the Institutional Review Board of Seoul National University Hospital (no. H-1803-044-926) and complied with the Declaration of Helsinki. This retrospective, observational study included patients who underwent renal transplantation at Seoul National University Hospital between 2000 and 2017. KT recipients with serum lipid profiles performed 1 year after transplantation were enrolled. There were no age restrictions, and patients who had undergone a second renal transplantation or simultaneous transplantation of other organs were also included. The patients who underwent renal transplantation at another hospital were excluded.

### Data collection and definitions

All data were obtained from the hospital’s electronic medical records. Demographic characteristics including age, sex, height, weight, and body mass index (BMI) were collected. The type of renal replacement therapy before transplantation was reviewed, and the duration of dialysis was calculated on the basis of the date of dialysis initiation. The donor type (living or deceased) was also recorded. The donor and recipient’s ABO and human leukocyte antigen (HLA) typing data were collected and compared to identify ABO incompatibility and the number of HLA mismatches. As our center utilizes a standardized, maintenance immunosuppressant regimen of steroids, mycophenolic acid, and calcineurin inhibitors with only a few exceptions, the prescription rate of steroids or mycophenolic acid was over 90% in the total cohort. However, the use of calcineurin inhibitors differed between patients; therefore, the type of calcineurin inhibitor was collected and analyzed. In addition, the use of mammalian target of rapamycin inhibitors was collected within 1 year after transplantation. The presence of comorbidities such as hypertension, diabetes mellitus (DM), and CVD before transplantation was analyzed. A history of CVD was defined as any history of heart failure, coronary artery disease, or cerebrovascular disease before transplantation. In addition, the duration of statin treatment after transplantation was reviewed. In our study, statin use was defined as the prescription of any 3-hydroxy-3-methyl-glutanyl-coenzyme A reductase inhibitor for at least 3 months during the first year after transplantation. In addition, use of fibrates and omega-3-acids has been defined as the prescription of any medication containing fibrates or omega-3-acids, respectively, for at least 3 months during the first year after transplantation. And steroid pulse therapy for rejection after transplantation was considered to be a factor affecting the lipid profile, so we reviewed the history of IV steroid pulse treatment within 1 year in study participants.

Total cholesterol, triglyceride, HDL-C, and LDL-C levels 1 year after renal transplantation were reviewed, and the TG/HDL-C ratio was calculated. In our center, patients are advised to measure the lipid profile after at least 12 h of fasting. Each lipid profile was categorized into five groups according to the values.

### Outcomes

The main outcome was the association between TG/HDL-C and MACE. MACE was defined as heart failure, coronary artery disease documented by coronary angiography, and cerebrovascular disease confirmed by imaging. All patients were followed until March 2018.

The secondary outcome was the association of TG/HDL-C with both graft and patient survival. Graft failure was defined as a return to dialysis or kidney re-transplantation. Mortality data were obtained from the National Database of Statistics Korea.

Biopsy findings during the first year after transplantation were also collected from the medical records. In our center, protocol biopsies were performed at time zero (post-reperfusion), on the 10th day, and 1 year after transplantation only if the patient agreed. Additional kidney biopsies were performed if graft function deteriorated or any suspicious symptoms or signs of rejection were observed. All pathologic findings were examined by two experienced nephropathologists.

### Statistical analyses

Data are expressed as mean ± standard deviation or counts with percentages. For comparisons of baseline categorical variables, the Chi-square and Fisher’s exact tests were used. Continuous variables were compared using analysis of variance. Survival analysis between TG/HDL-C and MACE, graft failure, and patient survival was assessed with univariate and multivariate Cox proportional hazard regressions. The proportional hazard assumption was checked for each multivariate Cox regression. The Stata function, mkspline, was used to create a restricted cubic spline function to describe the hazard ratio (HR) of MACE according to lipid profiles including TG/HDL-C. All statistical analyses were performed with STATA/MP version 15.1 (StataCorp, College Station, TX, USA). A *p* value of 0.05 was considered to indicate statistical significance in all tests.

## Results

### Baseline characteristics of study patients

A total of 1301 KT recipients were enrolled in the final analysis. The mean patient age was 40.9 ± 16.2 years, and men comprised 61.7% of the study population. The statin prescription rate was 30.3%. The patients showed total cholesterol level of 177.3 ± 33.2 mg/dL, TG of 128.5 ± 75.0 mg/dL, and HDL-C of 56.7 ± 16.7 mg/dL at 1 year after transplantation. LDL-C was also reviewed but only 785 of the 1301 (60.3%) patients had results, and the mean level of LDL-C was 96.4 ± 27.5 mg/dL. Table 1 reports the baseline characteristics of all patients.

**Table 1:** Baseline characteristics of the patient population

Variables	*n* = 1301
Age, years	40.9 ± 16.2
Male sex, *n* (%)	803 (61.7)
BMI, kg/m^2^	22.1 ± 3.9
Donor type, *n* (%)	
Living donor	876 (67.3)
Deceased donor	425 (32.7)
RRT type, *n* (%)	
Pre-emptive	207 (15.9)
Hemodialysis	780 (60.0)
Peritoneal dialysis	314 (24.1)
Duration of RRT, months	36.6 ± 44.8
ABO incompatible renal transplantation, *n* (%)	75 (5.8)
HLA mismatch, *n* (%)	
≤ 3	810 (62.3)
> 3	491 (37.7)
Type of calcineurin inhibitor, *n* (%)	
None	48 (3.7)
Cyclosporine	200 (15.4)
Tacrolimus	1053 (80.9)
mTOR inhibitor in 1 year after transplant, *n* (%)	58 (4.5)
Prior history of CVD, *n* (%)	77 (5.9)
HTN, *n* (%)	1164 (89.5)
DM, *n* (%)	379 (29.1)
Use of statins, *n* (%)	394 (30.3)
Steroid pulse for rejection within 1 year after transplant	569 (43.7%)
Lipid profiles at 1 year post-transplantation	
Total cholesterol (mg/dL)	177.3 ± 33.2
TG (mg/dL)	128.5 ± 75.0
HDL-C (mg/dL)	56.7 ± 16.7
†LDL-C (mg/dL)	96.4 ± 27.5

*BMI* body mass index, *RRT* renal replacement therapy, *HLA* human leukocyte antigen, *mTOR* mammalian target of rapamycin, *CVD* cardiovascular disease, *HTN* hypertension, *DM* diabetes mellitus, *TG* triglyceride, *HDL-C* high density lipoprotein cholesterol, *LDL-C* low density lipoprotein cholesterol

^†^Only available in 785 (60.3%) patients

### Risk factors associated with MACE

The median follow-up duration after renal transplantation in the total study population was 7.4 years (interquartile range 4.4–11.1 years). During the follow-up period, 80 (6.2%) patients developed MACE, which included 38 of unstable anginas, 9 of MIs, 19 of heart failures, 18 of cerebral infarcts, and 4 of cerebral hemorrhages. The total number of events was 89.

Then, we explored risk factors for development of MACE after renal transplantation. In the univariate analysis, age over 60 years, male sex, BMI over 25 kg/m^2^, deceased donor, prior history of CVD, hemodialysis as the RRT modality before transplant, duration of RRT over 1 year, high numbers of HLA mismatch, a history of diabetes and statin usage were related to an increased HR of MACE. However, after adjustment for multiple variables including lipid profiles, aged over 60 years and a history of diabetes showed a statistically significant elevation of the HR for MACE (Table 2).

**Table 2:** Univariate and multivariate cox analysis for MACE between categorical baseline characteristics and quintiles of each lipid profiles

Variables (Quintiles)		Levels (mean ± SD)		Univariate		†Multivariate	
				HR (95% CI)	*p*	HR (95% CI)	*p*
Age ≥ 60 years				3.79 (2.26–6.35)	< 0.001	2.36 (1.33–4.20)	0.003
Female				0.58 (0.36–0.95)	0.030	0.69 (0.41–1.17)	0.168
BMI ≥ 25 kg/m^2^				1.96 (1.20–3.21)	0.007	1.19 (0.71–2.01)	0.514
Deceased donor				1.62 (1.04–2.54)	0.034	1.10 (0.65–1.87)	0.713
Prior history of CVD				3.60 (1.98–6.54)	< 0.001	1.31 (0.67–2.55)	0.432
RRT type							
Pre-emptive				1 (reference)		1 (reference)	
Hemodialysis				2.76 (1.19–6.39)	0.018	2.02 (0.81–5.08)	0.133
Peritoneal dialysis				1.45 (0.56–3.77)	0.449	1.18 (0.41–3.41)	0.758
Duration of RRT ≥ 1 year				1.65 (1.03–2.62)	0.036	1.24 (0.70–2.19)	0.463
ABO incompatible KT				0.70 (0.17–2.89)	0.626		
HLA mismatch > 3				1.61 (1.04–2.50)	0.034	1.26 (0.79–2.00)	0.335
Type of calcineurin inhibitor							
None				1 (reference)			
Cyclosporine				0.39 (0.12–1.26)	0.115		
Tacrolimus				0.73 (0.26–1.99)	0.533		
mTOR inhibitor				1.07 (0.34–3.40)	0.910		
HTN				1.52 (0.47–4.87)	0.485		
DM				3.86 (2.46–6.03)	< 0.001	2.96 (1.85–4.75)	< 0.001
Use of statins				1.71 (1.08–2.70)	0.021	1.45 (0.89–2.36)	0.137
Steroid pulse for rejection				1.25 (0.81–1.94)	0.319		
Total cholesterol							
Q1		132.7 ± 14.3		1 (reference)		1 (reference)	
Q2		159.6 ± 5.5		0.72 (0.36–1.45)	0.360	0.82 (0.40–1.71)	0.601
Q3		176.1 ± 4.1		0.94 (0.49–1.82)	0.865	1.04 (0.50–2.16)	0.912
Q4		191.8 ± 5.5		0.46 (0.21–1.03)	0.061	0.43 (0.18–1.03)	0.059
Q5		225.0 ± 22.4		1.12 (0.60–2.10)	0.727	1.07 (0.50–2.28)	0.856
TG							
Q1		62.0 ± 9.8		1 (reference)			
Q2		87.7 ± 6.3		1.32 (0.63–2.78)	0.465		
Q3		111.1 ± 7.6		1.00 (0.46–2.15)	0.990		
Q4		143.3 ± 12.0		1.58 (0.78–3.20)	0.206		
Q5		236.7 ± 97.3		1.70 (0.85–3.41)	0.138		
HDL-C							
Q1		36.0 ± 4.8		1 (reference)		1 (reference)	
Q2		46.8 ± 2.3		0.75 (0.40–1.41)	0.371	1.01 (0.50–2.03)	0.982
Q3		54.3 ± 2.2		0.65 (0.34–1.23)	0.189	1.00 (0.47–2.16)	0.992
Q4		63.3 ± 2.9		0.54 (0.27–1.09)	0.085	0.89 (0.35–2.26)	0.800
Q5		80.9 ± 11.9		0.50 (0.25–1.00)	0.051	0.95 (0.32–2.83)	0.934
‡LDL-C							
Q1		61.9 ± 11.2		1 (reference)			
Q2		81.0 ± 3.9		0.66 (0.26–1.71)	0.397		
Q3		93.7 ± 3.6		0.79 (0.32–1.96)	0.607		
Q4		108.3 ± 4.3		0.86 (0.35–2.07)	0.731		
Q5		135.9 ± 20.3		0.77 (0.31–1.93)	0.582		
TG/HDL-C							
Q1		0.9 ± 0.2		2.21 (0.90–5.42)	0.084	2.49 (1.01–6.23)	0.047
Q2		1.5 ± 0.1		1 (reference)		1 (reference)	
Q3		2.0 ± 0.2		1.89 (0.75–4.74)	0.175	1.77 (0.71–4.45)	0.223
Q4		2.9 ± 0.3		3.41 (1.46–7.96)	0.004	3.38 (1.44–7.95)	0.005
Q5		5.8 ± 3.9		3.31 (1.41–7.75)	0.006	2.67 (1.13–6.30)	0.025

*HR* hazard ratio, *BMI* body mass index, *RRT* renal replacement therapy, *KT* kidney transplantation, *HLA* human leukocyte antigen, *mTOR* mammalian target of rapamycin, *CVD* cardiovascular disease, *HTN* hypertension, *DM* diabetes mellitus, *TG* triglyceride, *HDL-C* high density lipoprotein cholesterol, *LDL-C* low density lipoprotein cholesterol, *TG/HDL-C* ratio of triglyceride to high density cholesterol

^†^Adjusted by variables which showed *p* value under 0.1 in univariate Cox analysis

^‡^For the cox regression analysis of LDL-C, only 785 patients included. And the multivariate analysis for LDL-C was performed with same adjustment variables with other multivariate analysis

**Fig. 1:**
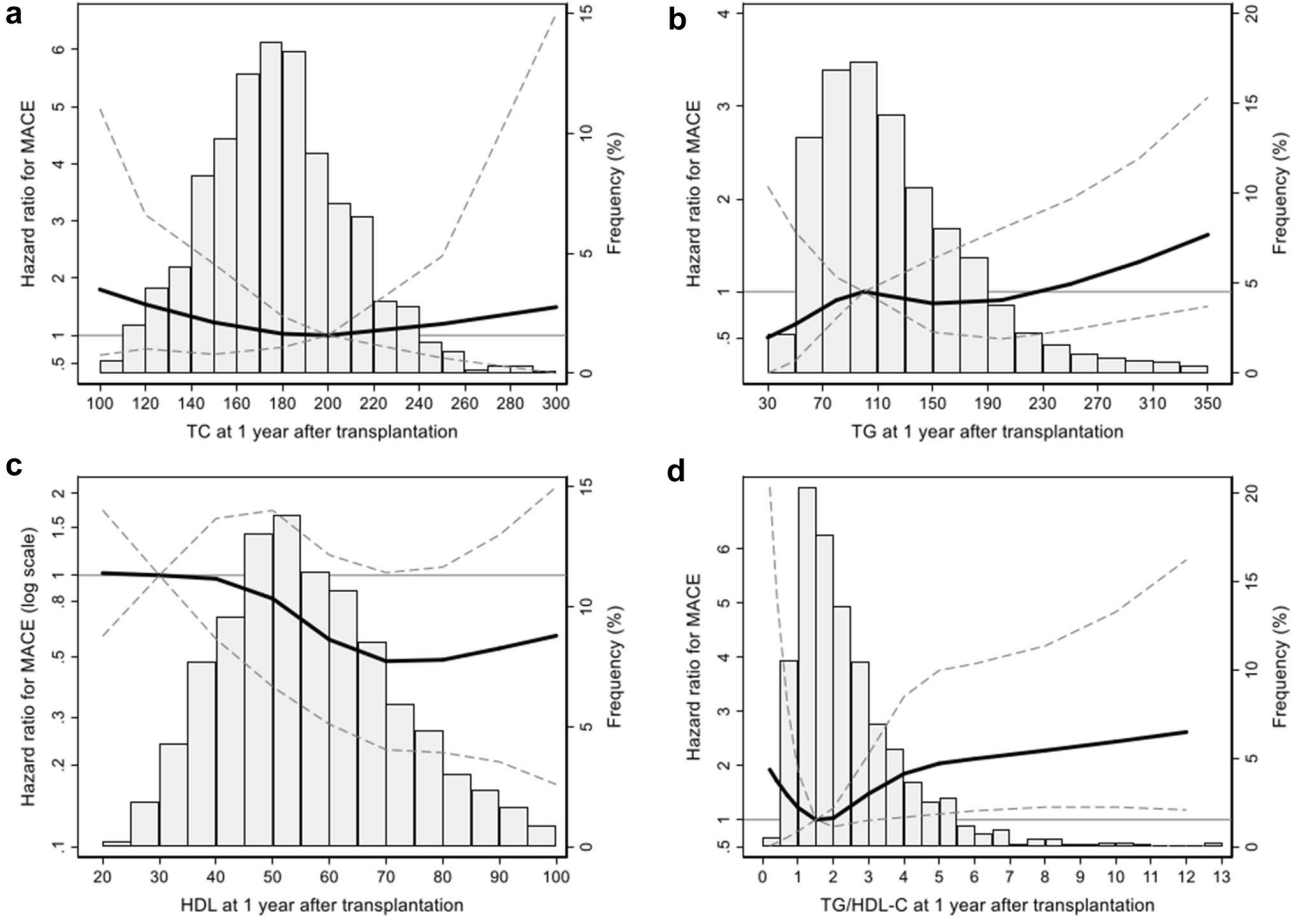
The multivariate-adjusted restricted cubic spline analysis by lipid profiles on MACE risk. Total cholesterol (**a**), triglyceride (**b**), HDL-C (**c**), and TG/HDL-C (**d**) were analyzed by multivariate adjusted cox regression and plotted with spline curve. The bar plots showed the frequency of patients on each lipid profile value. The thick solid line showed hazard ratio and the dashed line represented 95% confidence interval. *HDL-C* high density lipoprotein cholesterol, *MACE* major adverse cardiovascular event, *TC* total cholesterol, *TG* triglyceride

For the lipid profile, cubic spline analysis was performed on the continuous variable before conducting cox regression on the categorical variable (Fig. 1). In the cubic spline analysis, among the lipid profiles, TC, TG, and HDL-C showed no significant tendency for risk of MACE according to lipid levels. Only TG/HDL-C showed significant correlation with MACE risk and a significant increase in MACE risk at high TG/HDL-C values. To perform cox regression analysis for these lipid profiles, the lipid profile was divided into five groups according to the values. The first quintile was defined as the reference group for the analysis, but in the case of TG/HDL-C, the second quintile was defined as the reference group because the spline curve showed J shape. In univariate and multivariate analyses, the fourth and fifth quintiles of TG/HDL-C showed a significant elevation of the HR compared to the reference group [fourth quintile: aHR, 3.38; 95% confidence interval (CI) 1.44–7.95; *p* = 0.005; fifth quintile: aHR, 2.67; 95% CI 1.13–6.30; *p* = 0.025]. Moreover, the first quintile showed marginal difference of MACE risk compared to the reference group (aHR, 2.49; 95% CI 1.01–6.23; *p* = 0.047). The cubic spline curves and results of cox regression are shown in Fig. 1 and Table 2, respectively.

**Fig. 2:**
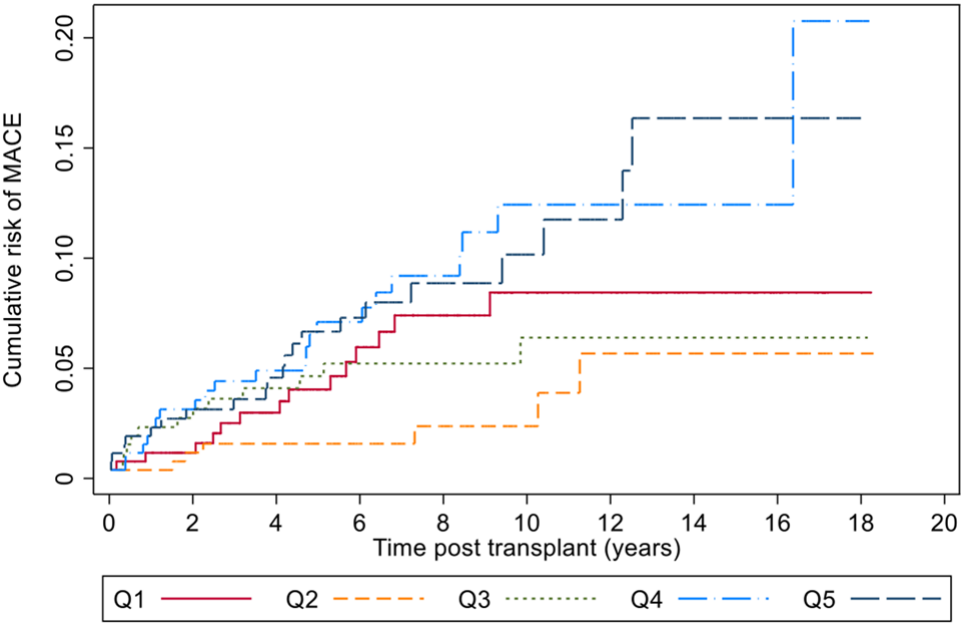
Cumulative risk for MACE in the quintiles of TG/HDL-C. The second quintile (yellow line) showed lowest risk and the fourth and fifth quintiles (light blue and blue lines) showed a significantly elevated risk compared to the second quintile of TG/HDL-C (log rank *p* = 0.003 for the fourth quintile, *p* = 0.004 for the fifth quintile). Q1-5 in the legends represent each quintile of TG/HDL-C

Nelson–Aalen cumulative hazard curves for quintiles of TG/HDL-C also showed a significant difference of cumulative risk at the fourth and fifth quintiles compared to the second quintile (log rank *p* = 0.003 for the fourth quintile, *p* = 0.004 for the fifth quintile). Figure 2 shows the cumulative risk for MACE in the quintiles.

### Subgroup analyses

When comparing the baseline characteristics of the TG/HDL-C quintiles, there were significant differences in the proportion of men, BMI, prior history of CVD and DM, and statin use. Higher TG/HDL-C quintiles had a higher ratio of men, a higher BMI, and a more frequent history of CVD and DM. Statin prescription rate was the lowest in the third quintile. Although there was no statistical significance, the statin prescription rate was higher in the fourth and fifth quintiles than in the first and second quintiles. Table 3 shows the demographic and clinical characteristics of each quintile of TG/HDL-C.

**Table 3:** Comparison of baseline characteristics and MACE events by the quintiles of TG/HDL-C

Variables	Q1	Q2	Q3	Q4	Q5	*p*
	*n* = 259	*n* = 261	*n* = 260	*n* = 259	n = 262	
Age, years	38.8 ± 17.0	40.5 ± 16.2	43.2 ± 16.1	39.7 ± 16.4	42.5 ± 15.0	0.008
Male sex, *n* (%)	113 (43.3)	140 (54.1)	168 (64.6)	185 (71.4)	197 (75.2)	< 0.001
BMI, kg/m^2^	20.7 ± 4.4	21.9 ± 3.2	22.3 ± 3.4	22.2 ± 4.2	23.3 ± 3.6	< 0.001
Donor type, *n* (%)						0.504
Living donor	172 (66.4)	167 (64.0)	173 (66.5)	178 (68.7)	186 (71.0)	
Deceased donor	87 (33.6)	94 (36.0)	87 (33.5)	81 (31.3)	76 (29.0)	
RRT type, *n* (%)						0.375
Pre-emptive	34 (13.1)	49 (18.8)	39 (15.0)	45 (17.4)	40 (15.3)	
Hemodialysis	157 (60.6)	155 (59.4)	157 (60.4)	143 (55.2)	168 (64.1)	
Peritoneal dialysis	68 (26.3)	57 (21.8)	64 (24.6)	71 (27.4)	54 (20.6)	
Duration of RRT, months	39.0 ± 47.3	40.5 ± 47.8	36.0 ± 42.6	34.9 ± 44.7	32.4 ± 41.2	0.242
ABO incompatible renal transplantation, *n* (%)	13 (5.0)	17 (6.5)	14 (5.4)	13 (5.0)	18 (6.9)	0.835
HLA mismatch > 3, *n* (%)	78 (30.1)	104 (39.8)	108 (41.5)	97 (37.5)	104 (39.7)	0.063
Type of calcineurin inhibitor, *n* (%)						0.152
None	7 (2.7)	5 (1.9)	11 (4.2)	13 (5.0)	12 (4.6)	
Cyclosporine	43 (16.6)	33 (12.6)	43 (16.5)	34 (13.1)	47 (17.9)	
Tacrolimus	209 (80.7)	223 (85.4)	206 (79.2)	212 (81.9)	203 (77.5)	
mTOR inhibitor in 1 year after transplant, *n* (%)	9 (3.5)	7 (2.7)	10 (3.8)	15 (5.8)	17 (6.5)	0.174
Prior history of CVD, *n* (%)	7 (2.7)	9 (3.4)	19 (7.3)	22 (8.5)	20 (7.6)	0.011
HTN, *n* (%)	228 (88.0)	237 (90.8)	231 (88.8)	228 (88.0)	240 (91.6)	0.597
DM, *n* (%)	60 (23.2)	67 (25.7)	82 (31.5)	80 (30.9)	90 (34.4)	0.032
Use of statins, *n* (%)	75 (29.0)	76 (29.1)	70 (26.9)	83 (32.0)	90 (34.4)	0.379
Steroid pulse for rejection within 1 year after transplant	99 (38.2)	114 (43.7)	116 (44.6)	115 (44.4)	125 (47.7)	0.287
Lipid profiles at 1 year post-transplantation, mg/dL						
Total cholesterol	178.3 ± 29.3	175.0 ± 29.3	176.9 ± 33.6	174.9 ± 36.0	181.7 ± 36.9	0.114
TG	67.5 ± 15.7	92.2 ± 20.0	114.5 ± 25.4	142.0 ± 32.1	225.5 ± 104.2	< 0.001
HDL-C	74.1 ± 16.2	62.8 ± 12.8	56.0 ± 12.1	49.5 ± 10.5	41.1 ± 9.4	< 0.001
TG/HDL-C	0.9 ± 0.2	1.5 ± 0.1	2.0 ± 0.2	2.9 ± 0.3	5.8 ± 3.9	< 0.001
Total number of MACE events, *n*	17	7	14	26	24	89
Coronary vascular	8	3	5	15	16	48
Cerebrovascular	6	0	4	6	6	22
Heart failure	3	4	5	5	2	19

*TG* triglyceride, *HDL-C* high-density lipoprotein cholesterol, *BMI* body mass index, *RRT* renal replacement therapy, *HLA* human leukocyte antigen, *mTOR* mammalian target of rapamycin, *CVD* cardiovascular disease, *HTN* hypertension, *DM* diabetes mellitus, *TG* triglyceride, *HDL-C* high density lipoprotein cholesterol

**Fig. 3:**
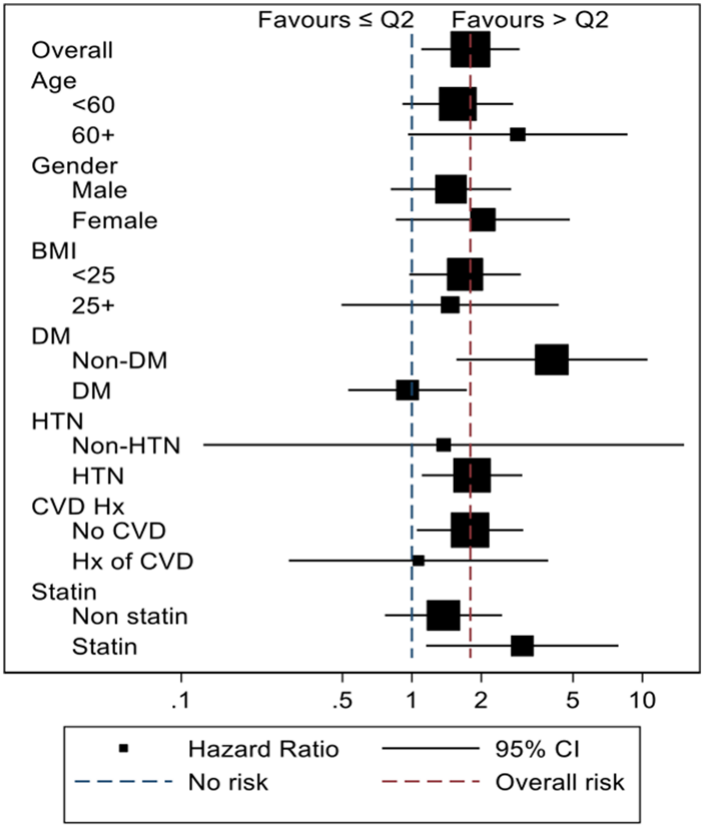
Subgroup analysis for baseline characteristics and effect of TG/HDL-C on MACE risk. Based on the blue dotted line, the left side shows an increase in MACE risk at low TG/HDL-C (less than the second quintile) and the right side shows an increase in MACE risk at high TG/HDL-C (more than the second quintile). The red dotted line shows the overall effect on MACE with TG/HDL-C

Subgroup analysis was performed to evaluate the confounding effect of multiple baseline characteristics (Fig. 3). For the subgroup analysis, we divided TG/HDL-C into two groups, the second quintile or lower and greater than the second quintile. This criterion is due to the lowest MACE risk by TG/HDL-C in the second quintile. When comparing the MACE risk between the two categories of TG/HDL-C, age, sex, and BMI showed no significant difference of risk between the subgroups. In contrast, the group with a history of diabetes showed no difference in risk according to TG/HDL-C, whereas the group with no history of diabetes showed an elevated risk of MACE. Similarly, the subgroups with a history of hypertension, no history of CVD, and statin usage were related to the risk of MACE; the counterparts of these subgroups showed no significant risk elevation.

### Association of TG/HDL-C with interstitial fibrosis and tubular atrophy, T-cell mediated rejection and the graft and patient survival

To determine the effect of TG/HDL-C on graft pathology, 1-year protocol biopsy results were collected. In our center, protocol biopsy was recommended to KT recipients 1 year after transplantation and performed if informed consent could be obtained. Approximately one-third of KT recipients (*n* = 387, 29.7%) underwent allograft biopsy 1 year after transplantation. Among these recipients, logistic analysis showed no significant relationship between TG/HDL-C and 1-year post-transplant interstitial fibrosis and tubular atrophy (IFTA) or T-cell mediated rejection (Table 4). Additionally, the cox regression analysis was performed on all study patients for graft and patient survival. We found lower risk of graft failure in the first quintile of TG/HDL-C compared to reference group with marginal significance (aHR, 0.48; 95% CI 0.22–1.03; *p* = 0.058, Table 4).

**Table 4:** IFTA in 1 year post-transplant kidney biopsy and the graft and patient survival according to quintiles of TG/HDL-C

TG/HDL-C	IFTA in 1 year post-transplant renal biopsy †*N* = 182/387		TCMR in 1 year post-transplant renal biopsy †*N* = 188/387		Graft failure *N* = 93/1301		Patient mortality *N* = 40/1301	
	Adjusted OR (95% CI)	*p*	Adjusted OR (95% CI)	*p*	aHR (95% CI)	*p*	aHR (95% CI)	*p*
Q1	0.73 (0.37–1.43)	0.359	0.62 (0.32–1.19)	0.151	0.48 (0.22–1.03)	0.058	0.48 (0.22–1.04)	0.064
Q2	1 (reference)	–	1 (reference)	–	1 (reference)	–	1 (reference)	–
Q3	1.57 (0.78–3.17)	0.210	0.81 (0.41–1.62)	0.556	0.93 (0.49–1.75)	0.815	1.02 (0.54–1.92)	0.955
Q4	1.35 (0.67–2.70)	0.398	1.08 (0.55–2.11)	0.828	1.22 (0.67–2.24)	0.518	1.38 (0.76–2.50)	0.297
Q5	1.30 (0.69–2.45)	0.423	0.99 (0.53–1.83)	0.973	1.88 (0.46–1.69)	0.698	0.99 (0.52–1.90)	0.986

*IFTA* interstitial fibrosis and tubular atrophy, *TCMR* T-cell mediated rejection, *TG* triglyceride, *HDL-C* high-density lipoprotein cholesterol, *OR* odds ratio

^†^Only included 1-year post transplant kidney biopsy findings which is available on medical records. Only formal pathologic results by nephropathologist was included

## Discussion

In this study, among the conventional lipid profiles and TG/HDL-C, TG/HDL-C was the only one that clearly correlated with MACE risk. MACE risk was significantly higher in the first, fourth and fifth quintiles of TG/HDL-C at 1 year after transplantation than the second quintile of TG/HDL-C. Subgroup analysis by baseline characteristics showed that the subgroups with non-DM, HTN, no history of CVD, and statin usage were related to elevated risk of MACE according to TG/HDL-C.

Increasing evidence has shown that small, dense LDL (sd-LDL) is the lipid most strongly linked to CVD, and its deregulation is the most frequent form of dyslipidemia in patients with premature heart disease [25]. sd-LDL easily penetrates the arterial wall, has a high affinity to the proteoglycans of the arterial wall, and has a long residence time in the subendothelial space [26]. In addition, it exhibits a low affinity for LDL receptors and is not easily removed from plasma [26]. Thus, sd-LDL particles might be a better predictor of CVD than traditional lipid profiles, including LDL-C. However, the direct measurement of sd-LDL is technically demanding and not applicable to routine biochemical screening [24]. A surrogate marker for sd-LDL is TG/HDL-C, which is increasingly being utilized because it is easy to measure. A growing body of research suggests that TG/HDL-C measurements can be used to predict CV risk [20, 23, 27]. Previous studies showed that TG/HDL-C is associated with extensive coronary disease and MI in the general population [23, 27, 28]. In the meta-analysis of Asia/Pacific region studies, total cholesterol/HDL-C and TG/HDL-C were found to be better predictors of CVD than other lipid variables [29]. In addition, TG/HDL-C is associated with CV risk in patients with chronic kidney disease and patients with obesity and type 2 DM [30, 31].

Moreover, a previous study reported disturbances in lipid metabolism associated with TG-rich lipoproteins and HDL particles in KT recipients, both of which are closely related to TG/HDL-C [32]. These changes in lipid profile indicate TG/HDL-C fluctuation and are presumed to be correlated with increased CV risk in transplant patients. However, despite this reasonable assumption, no previous studies have investigated TG/HDL-C in transplant patients.

In this study, we analyzed the MACE risk for each quintile of conventional lipid profiles, but none of the lipid profile showed significant correlation with MACE. Only the TG/HDL-C ratio, which combined TG and HDL-C, showed a significant change in MACE risk with changing quintiles. This specific significance of TG/HDL-C in MACE risk of KT recipients suggests that qualitative information including the morphological characteristics of cholesterol is more useful to predict CV risk than the quantitative amount of cholesterol.

The optimal cut-off value of TG/HDL-C remains unclear. However, a study in Iranian men indicated an increase in CV risk in the group with TG/HDL-C > 6.9 [33], and another study with a female cohort indicated an increase in CV risk in the group with TG/HDL-C > 3.66 [34]. In a study of patients with stage 1–5 chronic kidney disease, the risk of CV events was elevated when TG/HDL-C was > 3.29 [30]. In our study, the fourth and fifth quintiles that had an elevated risk of MACE showed TG/HDL-C of 2.9 ± 0.3 and 5.8 ± 3.9, respectively. According to the result of our study, the cut-off value of TG/HDL-C in KT recipients could be 2.9 or above, which is similar to those in prior studies on chronic kidney disease.

When comparing the baseline characteristics according to the quintile of TG/HDL-C, the male ratio, BMI, CV history, and DM were higher in the fourth and fifth quintiles. The differences in these baseline characteristics were thought to affect MACE risk; therefore, a stratified subgroup analysis was performed according to the baseline characteristics. In the subgroup analysis, among the characteristics that differed between quintiles, the sex and BMI subgroups showed no difference in MACE risk according to TG/HDL-C, whereas the CV history and DM subgroups showed a significant difference in MACE risk according to TG/HDL-C. The possible cause of the differing influence of TG/HDL-C between these subgroups is that patients with high risk factors such as DM and CVD history before transplant are more affected by such risk factors than TG/HDL-C alone, so that there is little difference in the risk for TG/HDL-C levels, while the patients without those risk factors can have TG/HDL-C as the significant risk factor of MACE.

We also found graft survival was marginally increased in the lowest quintile of TG/HDL-C compared to the reference group. Several previous studies showed evidence of relationship of lipid profile on graft survival. One study showed hypertriglyceridemia correlates with chronic allograft nephropathy [35], and another study showed that the combination of triglyceride and VLDL proteins correlate with graft failure [36]. Although the results of our study on graft survival did not gain statistical significance, we could suggest lipid profile ratio as well as conventional profile might affect the graft outcome in KT recipients.

The present study has several limitations. First, this was a retrospective observational study; therefore, it is difficult to prove cause and effect. Second, the TG/HDL-C index, which we used, was calculated using two lipid profiles, TG and HDL-C, and TG may have changes according to quantity of daily meal intake and measurement time after food intake. Although we used the lipid profile measured during fasting, nevertheless, it is possible that the TG/HDL-C group was misclassified in some patients due to diurnal and daily variation. Third, this study was conducted in a single country and included an exclusively Asian patient population. Other ethnic populations may exhibit differences under similar conditions. Finally, there is a possibility of selection bias, as only patients with a lipid profile at 1 year after transplantation were included in the study.

## Conclusion

In this study, we found a significant association between post-transplant TG/HDL-C and the development of MACE in KT recipients. Based on our findings, maintaining adequate TG/HDL-C levels in KT recipients may help to reduce CV risk and increase long-term graft survival.
